# Horizontal gene transfer of epigenetic machinery and evolution of parasitism in the malaria parasite Plasmodium falciparum and other apicomplexans

**DOI:** 10.1186/1471-2148-13-37

**Published:** 2013-02-11

**Authors:** Sandeep P Kishore, John W Stiller, Kirk W Deitsch

**Affiliations:** 1 Department of Microbiology and Immunology, Weill Cornell Medical College, New York, NY 10065, USA; 2 Department of Biology, East Carolina University, Greenville, NC, 27858, USA

**Keywords:** Protozoa, *Plasmodium*, Apicomplexa, Transcription, Parasitism, SET domain, Horizontal gene transfer

## Abstract

**Background:**

The acquisition of complex transcriptional regulatory abilities and epigenetic machinery facilitated the transition of the ancestor of apicomplexans from a free-living organism to an obligate parasite. The ability to control sophisticated gene expression patterns enabled these ancient organisms to evolve several differentiated forms, invade multiple hosts and evade host immunity. How these abilities were acquired remains an outstanding question in protistan biology.

**Results:**

In this work, we study SET domain bearing genes that are implicated in mediating immune evasion, invasion and cytoadhesion pathways of modern apicomplexans, including malaria parasites. We provide the first conclusive evidence of a horizontal gene transfer of a Histone H4 Lysine 20 (H4K20) modifier, Set8, from an animal host to the ancestor of apicomplexans. Set8 is known to contribute to the coordinated expression of genes involved in immune evasion in modern apicomplexans. We also show the likely transfer of a H3K36 methyltransferase (Ashr3 from plants), possibly derived from algal endosymbionts. These transfers appear to date to the transition from free-living organisms to parasitism and coincide with the proposed horizontal acquisition of cytoadhesion domains, the O-glycosyltransferase that modifies these domains, and the primary family of transcription factors found in apicomplexan parasites. Notably, phylogenetic support for these conclusions is robust and the genes clearly are dissimilar to SET sequences found in the closely related parasite *Perkinsus marinus*, and in ciliates, the nearest free-living organisms with complete genome sequences available.

**Conclusions:**

Animal and plant sources of epigenetic machinery provide new insights into the evolution of parasitism in apicomplexans. Along with the horizontal transfer of cytoadhesive domains, O-linked glycosylation and key transcription factors, the acquisition of SET domain methyltransferases marks a key transitional event in the evolution to parasitism in this important protozoan lineage.

## Background

The evolutionary processes whereby free-living or symbiotic organisms made the transition to full-fledged obligate, intracellular parasites remain unclear. This is perhaps best exemplified by the case of the Apicomplexa, a group of ~5000 species that includes several major human disease-causing agents including *Toxoplasma gondii*, *Cryptosporidium spp*. and *Plasmodium falciparum*, the most lethal of human malaria parasites [1]. Presumably, this transition involved the development of novel cellular differentiation pathways that enabled infection and replication within different hosts, invasion schema, cytoadhesion to host substrates and immune evasion strategies including antigenic variation.

The newly acquired lifestyle complexity might also require the acquisition of new mechanisms to control gene expression. For example, more sophisticated transcriptional regulation and epigenetic machinery would enable the evolution of complex life cycles involving multiple hosts and stages, and facilitate developmental changes accompanying the transition to parasitism. This could involve either de novo innovations or the horizontal acquisition of transcriptional and epigenetic machinery from other eukaryotes. Examples of both mechanisms of innovation have been reported. For instance, we previously described the unusual and rapid evolution of the C-terminal domain of RNA polymerase II within the *Plasmodium* lineage, and more specifically the expansion of this domain in parasites that infect primates [2]. This domain is crucial for controlling many aspects of gene expression, including epigenetic mechanisms of control, and the rapid de novo evolution of host-specific modifications demonstrates how important aspects of gene expression are for parasitism.

In contrast, it is now well established that the primary class of transcription factors in apicomplexan parasites (the ApiAP2 family) was acquired through an ancient horizontal gene transfer (HGT) event [3]. It is known that the ancestor to apicomplexans engulfed an alga, endowing it with photosynthetic abilities and enabling it to synthesize several important products, including fatty acids [4,5]. The engulfed alga later degenerated into a relic, the apicoplast; this accompanied the loss of photosynthetic ability and the evolution of apicomplexans into obligate intracellular parasites metabolically dependent on their animal hosts. The ApiAP2 class of transcription factors was acquired horizontally from the relic alga and now contributes to controlling parasite gene expression. Similarly, protein domains involved in cytoadhesion and O-linked glycosylation appear to have been acquired through HGT, in these cases from the parasites’ hosts rather than the algal endosymbiont [6,7]. The origin of the innate immune system in early animals, the likely hosts of newly evolved apicomplexan parasites, also dates to this evolutionary period. Thus, the hallmarks of parasitism, including nutritional dependence, invasion, and immune escape likely developed as part of the same evolutionary process during which photosynthetic ability was lost.

In the case of the Apicomplexa, therefore, two major potential sources for HGT have been established: *i*) transfer from an algal endosymbiont (fatty acid synthases or ApiAP2 transcription factors) [3,8], or *ii*) transfer from an animal host (domains involved in cytoadhesion and O-linked glycosylation) [6,7]. Here, we consider the putative acquisition of epigenetic machinery in the ancestor of apicomplexans with a focus on histone lysine modifiers, which are central to pathways of cellular differentiation, cell invasion and immune evasion in apicomplexan parasites.

Histone lysine methyltransferases, characterized by a SET domain, play a fundamental role in gene activation and epigenetic regulation across all eukaryotes [9]. These domains modify histone lysine residues at Histone H3 Lysine 4, 9, 36, and Histone H4 Lysine 20. These modifications are crucial for the establishment and maintenance of epigenetic memory, including in *P. falciparum,* and are involved in imprinting genes involved in invasion and immune evasion [10-15]. Among these SET domain bearing modifiers, the epigenetic modifier Set8 is known to participate in mitosis and is thought to facilitate the transmission of heterochromatic marks through the cell cycle in higher eukaryotes as well as in the Apicomplexa [16]. In recent work, Sautel et al. (2007) sampled a small set of animal sources and found strong homology to apicomplexan Set8, raising the possibility that this gene was acquired by an ancient apicomplexan ancestor from its eukaryotic host. That study, however, did not explicitly address the likelihood of HGT or, if such an event did transpire, when in the course of apicomplexan evolution it likely occurred.

An extensive examination of SET domain containing proteins (and the corresponding demethylases) found in the *P. falciparum* genome was reported by Cui et al. [17]. One of these genes displays significant similarity to Set2, a chromatin modifier known to deposit methyl groups during active transcription by RNA polymerase II [18]. This protein is present in primate malaria parasites but conspicuously missing in the closely related rodent parasites. In this work, we provide evidence for horizontal transfer of these methyltransferases. Intriguingly, the proposed transfer events occur on the branch of the phylogenetic tree on which the parasitic lifestyle of apicomplexans appears to have evolved, including the acquisition of cytoadhesion domains and their O-glycosyltransferase modifiers. The acquisition of these various capabilities were essential steps in equipping these organisms to their new host-dependent lifestyles and therefore marks a key transitional event in the evolution to parasitism in this important protozoan lineage.

## Results

### Apicomplexan Set8 is derived from animal Set8

The histone methyltransferase Set8 of apicomplexan parasites was recently shown to display strong sequence similarity to the orthologous protein in animals [16], suggesting the possibility of an ancient horizontal acquisition of the gene encoding this protein. Therefore, we were interested in more extensive phylogenetic analyses of the origins of apicomplexan Set8. Using a canonical SET domain from *Homo sapiens* Set8 (PR-7) as the query, we searched for the closest sequences to the Set8 domain across all major eukaryotic phyla. In many organisms, no conclusive Set8 ortholog could be identified, and in all taxa other than animals and apicomplexans, sequences retrieved using human Set8 as query proved to be most similar to other Set families in reciprocal blast searches of the complete NCBI protein database, suggesting that they might not be bona fide Set8 orthologs. Nonetheless, to avoid erroneously excluding highly divergent sequences, the nearest blastp matches to human Set8 were included in our analyses. Our complete dataset therefore represented potential Set8 sequences from all major eukaryotic lineages for use in phylogenetic analyses.

**Figure 1:**
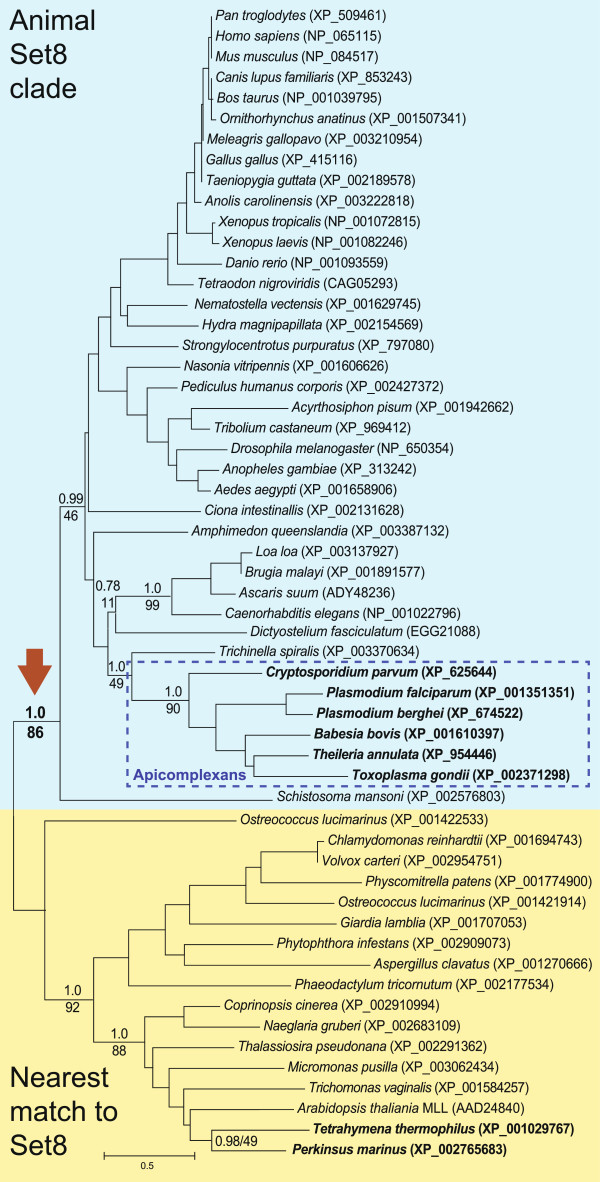
**Phylogenetic tree of eukaryotic organisms based on Set8 orthologs. **This tree based on Set8 shows a phylogenetic incongruence with respect to trees recovered by broad scale eukaryotic phylogenomics. Displayed in blue is the animal clade of Set8 denoted by a strongly supported node (large arrow). Apicomplexan Set8 groups within the metazoan/animal clade with strong posterior probability support that is unexpected given accepted eukaryotic relationships. Set8 is absent in other lower eukaryotes based on current genome sequences. Where the closest Set8 hits were found, the proteins group together in the yellow shaded box (with strong support). These are likely not Set8 as *Tetrahymena*, a representative ciliate known to lack Set8, is featured in this clade. Based on these observations, it is likely that the apicomplexan Set8 is derived from animals. A horizontal gene transfer provides the simplest, most parsimonious explanation.

A phylogenetic tree was derived from combined maximum-likelihood (ML) and Bayesian inference using Set8 orthologs across this broad sampling of eukaryotic organisms with the results shown in Figure 1. This tree differs significantly with current consensus phylogenies derived from larger datasets [19], which clearly show the great evolutionary distances between apicomplexans and the animal and plant kingdoms. Specifically, we recovered apicomplexan Set8 sequences as a monophyletic grouping that does not occur in the expected position on the tree. Instead of grouping with closely related protists such as *Perkinsus marinus* and ciliates [19], apicomplexan Set8 sequences nest strongly within the animal Set8 clade. Consistent with this, no other phyla or taxa show any phylogenetic affinity for apicomplexan Set8; this includes algal and plant sequences, which represent alternative potential sources for HGT. Moreover, the other SET domain bearing sequences (the lower clade in Figure 1) do not appear to represent bona fide Set8 proteins. *Tetrahymena* proteins are found in this clade, for instance, and *Tetrahymena* is known to lack Set8 [16]; as noted above, none of these sequences retrieved Set8 when used as queries in reciprocal blastp searches.

The recent completion of the *P. marinus* genome provided a particularly valuable resource for investigating whether the Set8 transfer likely occurred specifically in the ancestor of extant apicomplexans. *Perkinsus marinus,* a parasite of oysters, is the nearest known relative of dinoflagellates and therefore serves as a representative sister group of apicomplexans for our analysis. Because dinoflagellates are predominantly free-living, *Perkinsus* must have evolved its parasitic lifestyle independently of the apicomplexans. The nearest SET domain bearing sequence to human Set8 from the *P. marinus* genome (*E* ≤ 10^-13^) did not return a match to Set8 when used as a query in a reciprocal blastp search, and nested deeply within the clade of non-Set8 sequences in phylogenetic analyses (Figure 1). While caution must always be used when concluding that a sequence is absent from any particular genome, the *P. marinus* genome database includes over 23,000 inferred proteins, suggesting a relatively complete data set for proteomic comparisons.

In an expanded survey of eukaryotes, including partial genomic resources available to date, blast queries with human Set8 failed to identify any significant hits (*E* ≤ 10^-5^) in other organisms closely related to the apicomplexans, namely dinoflagellates, colpodellids, and *Chromera velia*, a photosynthetic autotroph discovered recently. The same was true for a variety of other protists including kinetoplastids, *Trichomonas* and *Giardia*, as well as fungi (including budding yeast), and also certain amoebozoans including *Acanthamoeba*.

As shown in Figure 1, statistical support for apicomplexan Set8 grouping with animals is very strong (Bayesian probability of 1.0 and ML bootstrap of 86%), while the closest sequences to Set8 in all other non-animal species grouped in a separate clade (also with very strong support). The only exception is the slime mold *Dictyostelium*, which also groups within the animal Set8 clade. Given that complete genomes of other members of the Amoebozoa phylum (e.g. *Entamoeba* and *Acanthamoeba*) lack Set8, this provides evidence of a similar horizontal transfer of animal Set8 into slime molds. Considering the vast evolutionary distances between animals, the Apicomplexa and slime molds, and the absence of Set8 from all other eukaryotic lineages, it appears almost certain that the latter two groups acquired Set8 independently via HGT from animals. The highly unlikely alternative would be direct descent of apicomplexan Set8 from a common ancestor with animals, followed by gene loss repeatedly and independently from all other eukaryotes except some cellular slime molds.

### A nematode appears to be the source of the host transfer to an ancient apicomplexan

The finding that apicomplexan Set8 is likely of animal origin raises questions about the approximate time of the transfer event, and the source of the acquired sequence. As noted above, the time of the transfer appears to be after the divergence of apicomplexans from dinoflagellates and the colopodellids [20] since these organisms appear to lack Set8, but prior to the radiation of the known apicomplexans, which all have Set8. To more accurately explore which specific animal taxon was the likely source of the gene, Set8 sequences from extant apicomplexans (*P. falciparum, T. annulata, T.gondii, C. parvum* and *B. bovis*) were used in phylogenetic analyses with only the animal and *Dictyostelium* sequences. To promote more accurate recovery of phylogenetic associations within the Set8 clade, we discarded highly divergent, non-orthologous SET sequences from other eukaryotes that could produce phylogenetic artifacts within the Set8 clade.

**Figure 2:**
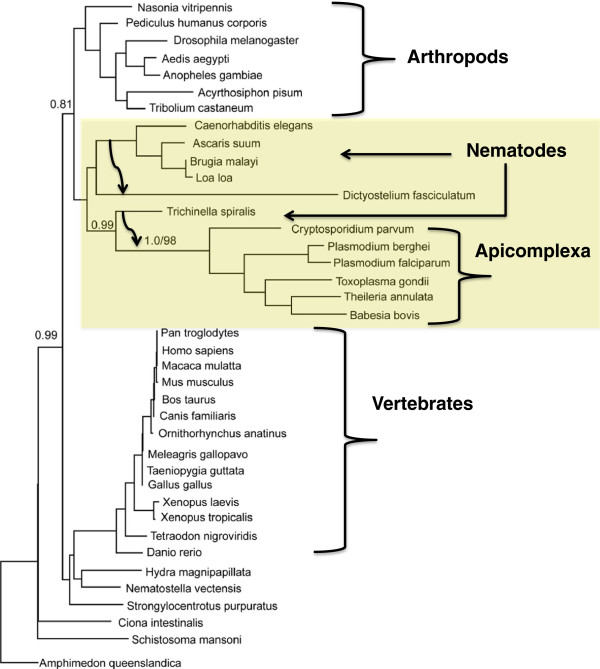
**A deeper look at Set8: Apicomplexa, animals and slime molds. **A phylogenetic analysis with only animal, apicomplexan and slime mold Set8. Set8 from Apicomplexa, nematodes and arthropods strongly group together (0.81 posterior probability) in a sub-clade apart from vertebrates. A Set8 ortholog from an animal, the parasitic nematode *Trichinella spiralis*, groups with strong Bayesian posterior probability with the Apicomplexans orthologs. Similarly, soil nematodes group with the soil-based slime mold *Dictyostelium*, implying a horizontal gene transfer event in this taxon as well. These proposed events are denoted by black arrows. As all apicomplexans studied feature Set8, the most likely scenario is a horizontal gene transfer to an ancestral apicomplexan.

As shown in Figure 2, we consistently observed that apicomplexan sequences branch within the nematodes, and specifically with Set8 from *Trichonella spiralis*. The ML bootstrap support for this relationship is weak (32%) but the Bayesian posterior probability is strong (0.99). Importantly, this tree recovers the same relationship between nematodes and Apicomplexa as did the expanded analysis, indicating this topology is stable regardless of the taxa sampled.

Furthermore, it is telling that both the slime mold *Dictyostelium* and the Apicomplexa branch with nematodes, but separate from each other. If the positions of the *Dictyostelium* and apicomplexan sequences were long-branch attraction artifacts, they would be expected to attract each other as the two most divergent branches of the Set8 tree. Moreover, neither is attracted to the base of the Set8 clade, or even to the base of the nematode clade, but rather to individual sub-clades within the nematodes. This contrasts with the clear artefactual rooting of the overall Set8 clade, wherein long-branch outgroups are attracted to the rapidly evolving sequence from the parasitic trematode *Schistosoma mansoni*. Thus, although statistical support for a specific relationship between apicomplexan and nematode sequences is not strong, there appears to be no basis for concluding it is a typical phylogenetic artefact. While the precise timing of the emergence of nematodes is a subject of debate, all estimates place their origin between 600– 1200 million years ago [21]. Thus, nematodes were extant at the time of the apicomplexan radiation, making the proposed transfer plausible.

### Analysis of the reciprocal H3K36 modifiers Set2 and JmjC1 in Apicomplexa

Similar to Set8, Set2 is a histone methyltransferase that contributes to the regulation of higher order chromatin assembly and the epigenetic control of gene expression. Set2 and its cognate demethylase JmjC1 work by adding and removing methyl groups from H3K36, respectively [22]. With the exception of the rodent plasmodia, all apicomplexan parasites, regardless of host, possess an apparently orthologous (within apicomplexans) protein similar to Set2. At least two ciliates (*Tetrahymena* and *Paramecium*) also possess putative Set2 homologs, suggesting that the Set2 family could have been present in the alveolate lineage before the divergence of the Apicomplexa from ciliates.

Aravind and colleagues have argued that a major family of transcription factors (ApiAP2) in Apicomplexa were laterally transferred from the algal endosymbiont harbored intracellularly by all members of this group of parasites [3]. This notion led us to question whether the chromatin modifiers Set2 and JmjC1 also could have originated from a similar horizontal transfer event. To determine if Set2 and JmjC1 might have been acquired from an algal endosymbiont, or any other higher eukaryotic cell, we performed bioinformatic and phylogenetic analyses as described previously for Set8.

### Horizontal transfer of a H3K36 modifier into the Apicomplexa

**Figure 3:**
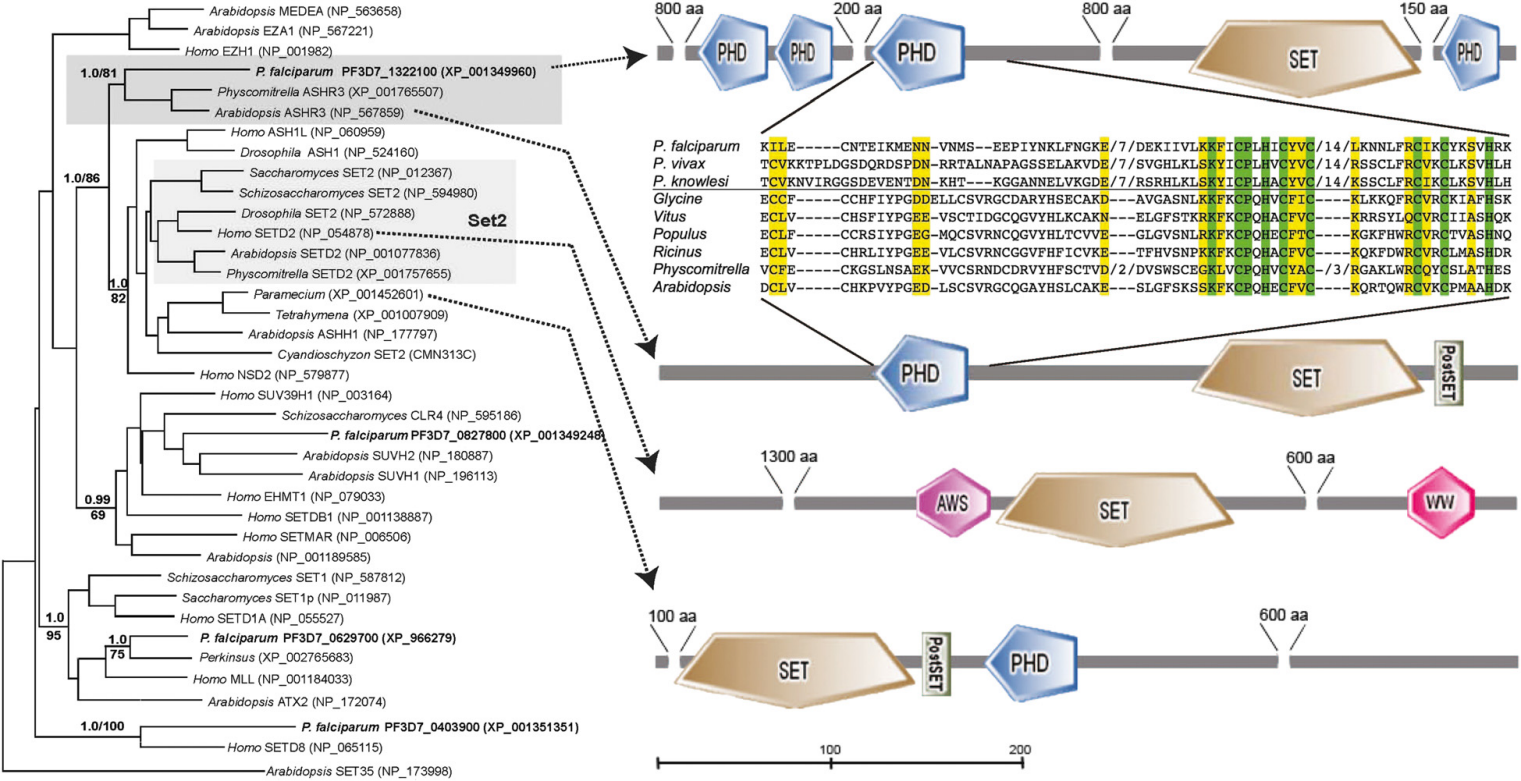
**Phylogenetic tree and domain characteristics of SET domain proteins from *P. falciparum. ***SET proteins were retrieved through reciprocal blastp searches and analyzed using maximum-likelihood and Bayesian inference. The upper, dark gray box indicates the strong grouping of *P. falciparum *‘Set2’ with Ashr3 sequences from green plants. The larger, lighter gray box below defines canonical Set2 sequences defined in model organisms. Although both are included in a larger clade of putative H3K36 modifying proteins, apicomplexan ‘Set2’ homologs do not group with their ostensible alveolate sister group, the ciliates (represented by *Tetrahymena *and *Paramecium*). The strong phylogenetic association with plant Ashr3 proteins is further supported by overall domain architecture, including (and unlike canonical Set2 and ciliate homologs) the shared C-terminal location of the SET domain, as well as a highly conserved PHD region that is proximal to the SET domain in both. In the alignment of this region included, residues that are invariable among plant and apicomplexan sequences are shaded green, those with conservative substitutions are in yellow. The only SET-containing sequence from *Perkinsus marinus *with significant similarity to Set2, is strongly supported as a member of the Set1 subfamily, and (as expected) as sister to the *P. falciparum *Set1 paralog.

In addition to Set8 discussed above, phylogenetic analyses clearly define *Plasmodium* proteins similar to the Set2 subfamily, as well as to Set1 and Set3 (Figure 3). Interestingly, although a Set2 subfamily including animal, yeast and higher plant exemplars is well resolved in our analyses (Figure 3), the putative Set2 sequence from *Plasmodium* (PF3D7_1322100) groups with Ashr3, a related H3K36 methyltransferase from green plants. An initial broader analysis performed with putative Set2 sequences from all apicomplexans recovered the same topology, but with weaker support (Additional file 1). To clarify the SET protein subfamily to which apicomplexan Set2 belongs, we produced a tree with more balanced sampling, using only sequences of *P. falciparum* along with Set family exemplars from plants, animals and yeast, and Set proteins from ciliates and *P. marinus*. The resulting tree is shown in Figure 3, and provides strong support in both ML and Bayesian analyses for an association between the *Plasmodium* sequence and Ashr3 homologs from green plants. In contrast, the nearest SET protein from *P. marinus*, the expected sister group to apicomplexans, is defined clearly as a member of the Set1 subfamily.

Because only the SET domain itself can be aligned across all these diverse sequences, a relatively short alignment (231 aa) is available for tree reconstruction; potentially this could lead to spurious phylogenetic associations. In this case, however, there is additional corroborating evidence outside the SET domain for a close relationship between PF3D7_1322100 and the plant Ashr3 family. In both cases, the SET domain is positioned at the C-terminal end of the protein. In contrast, Set2 orthologs from animal, yeast and green plant models have a SET domain in more N-terminal locations [23]. Potential Set2 homologs from ciliates, the nearest relatives of apicomplexans present in the Set2 sub-clade, also have SET domains positioned at their extreme N-termini. More significantly, Simple Modular Architecture Research Tool (SMART; [24]) identifies a shared plant homeodomain (PHD) as the first identifiable domain upstream of the SET domain (Figure 3). Although the complete gene from *Plasmodium* has undergone a dramatic expansion, this PHD region is conserved strongly enough between plant Ashr3 and *Plasmodium* genes to be found in reciprocal blastp searches; in contrast, no comparable match is found among numerous PHD and other ring domains in various SET proteins in the NCBI database.

The combination of this conserved PHD in comparable synteny with the SET domain, similar overall domain architecture, and strong phylogenetic support for SET domain monophyly between *Plasmodium* and plant Ashr3 are unlikely to be coincidental. Rather, they represent credible evidence of an orthologous relationship. The Ashr3 Set subfamily is not found in animals or fungi [23] and, based on our survey of complete NCBI protein databases, appears to be restricted to green plants and apicomplexans. Although we were unable to identify Ashr3 genes in either green or red algae, they are present in early land plants, and phylogenetic analyses, both ours and previous [23], indicate that Ashr3 is a relatively ancient SET protein family. In addition, all apicomplexan Ashr3/Set2 sequences are recovered as a monophyletic group (Additional file 1), indicating a single transfer event before the radiation of extant apicomplexans. Therefore, it is possible that Ashr3 was present in the algal ancestor of the apicoplast and moved into apicomplexans via endosymbiotic gene transfer.

At present, whether the apicoplast is descended from the same endosymbiont that gave rise to other photosynthetic alveolates remains under debate, as does the taxonomic affiliation of that algal endosymbiont [25,26]. Given this uncertainty, and the relative paucity of genomic data from dinoflagellates and red algae (a possible source of alveolate plastids), HGT of Ashr3 from an algal endosymbiont is purely speculative at this juncture, and remains one of several possibilities for the acquisition of this protein. Nevertheless, the known presence of an algal endosymbiont in the ancestor of apicomplexans provides a reasonable biological explanation for the presence of a plant-specific chromatin remodeling protein in the lineage. Whatever the vector, the horizontal transfer appears to predate the origin of extant apicomplexans, as *Cryptosporidum* contains a four-PHD SET protein that groups with PF3D7_1322100 and all related apicomplexan sequences in expanded phylogenetic analyses (Additional file 1).

**Figure 4:**
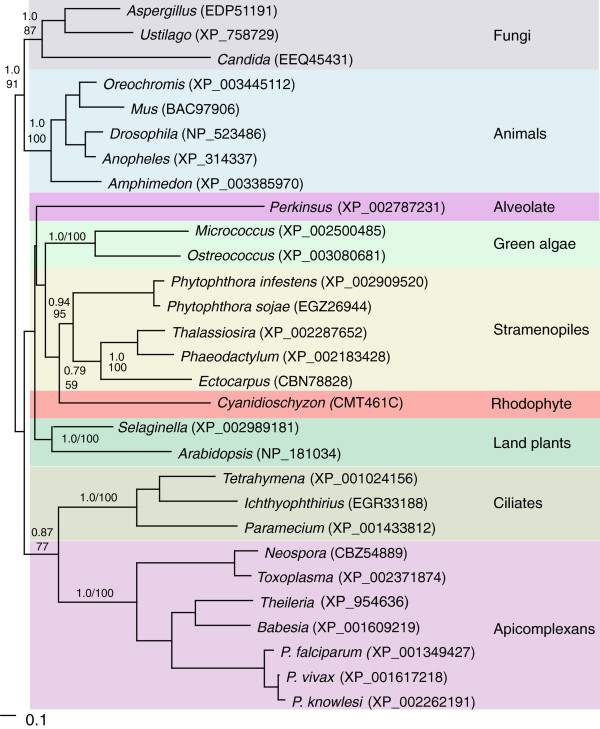
**Phylogenetic tree of JmjC1. **JmjC1 domains retrieved using *P. falciparum *and *T. gondii *orthologs as queries, and analyzed phylogenetically using Bayesian inference and maximum-likelihood. Support values for the respective analyses are shown above or to the right of key nodes important to the relative positions of apicomplexan, ciliates and potential sources of HGT. Although alternative sampling strategies, and inclusion of resulting exemplars from various green algae, separated ciliate and apicomplexan sequences in other phylogenetic analyses, in no case was phylogenetic support for a scenario of HGT as strong as support for the ciliate-apicomplexan sister relationship on this tree. Sequences from additional organisms could alter this tentative conclusion, however, given that JmjC1 from *Perkinsus*, an organism believed to be much closer than ciliates to apicomplexans, branches separately from other alveolates.

We also completed similar analyses for JmjC1 homologs (Figure 4). In this case, however, we recovered an apicomplexan association with sequences from ciliates, although not with the sequence from *P. marinus*. Although support for these relationships is reasonably strong, we should point out that it depends on how JmjC1 genes are sampled. For the analyses shown in Figure 4, we chose the closest blastp match to JmjC1 from *P. falciparum* or *T. gondii* from each taxon. In contrast, when we sampled sequences using human and yeast exemplars as queries, phylogenetic analyses tended to group apicomplexan JmjC1 with sequences from green algae. Nevertheless, we could find no compelling evidence at present to indicate that the history of JmjC1 in apicomplexans is complicated by HGT.

## Discussion

The putative horizontal transfer of nematode derived Set8 genes to both slime molds and an ancestor of apicomplexans is both interesting and biologically plausible. Both nematodes and arthropods (the next closest taxon in Set8 trees) were extant at the time the ancestor of apicomplexans acquired the Set8 ortholog. Whereas arthropods are known definitively as hosts for many apicomplexans today, apicomplexan parasitism of nematodes has not been documented (perhaps because parasites of nematodes have been under-studied). Although *Trichenella* is a modern parasite of mammals, the transfer of Set8 to an ancestral apicomplexan during co-infection of a mammalian host is highly unlikely, given that mammals had not yet appeared at the proposed time of the acquisition. It is thus more likely that the ancestral apicomplexan parasitized nematodes and acquired Set8 in a gene transfer event. The timing of this transfer event is more precisely defined through comparison to *P. marinus.* The absence of Set8 in the complete genome of this sister taxon to apicomplexans adds confidence to the proposal of a transfer event that took place *after* the divergence of apicomplexans.

What is the functional consequence of this transfer? Given the adaptations of apicomplexans toward intracellular niches, the acquisition of a major epigenetic regulator that governs and faithfully affirms chromatin-silencing marks could have been a critical step by protozoans for parasitism in diverse hosts with increasingly complex immune systems. H4K20Me1, the epigenetic mark deposited on histones by canonical Set8, is known to participate in gene silencing, heterochromatin formation and mitotic regulation as well as DNA damage checkpoint repair [27-29]. We propose that the acquisition of Set8 enabled higher order gene regulation, including cellular differentiation (for invading different hosts) as well as for animal immune system evasion. Acquisition of this type of regulation would also enable the evolution of complex life cycles that involve multiple hosts as well as dormant cyst stages.

**Figure 5:**
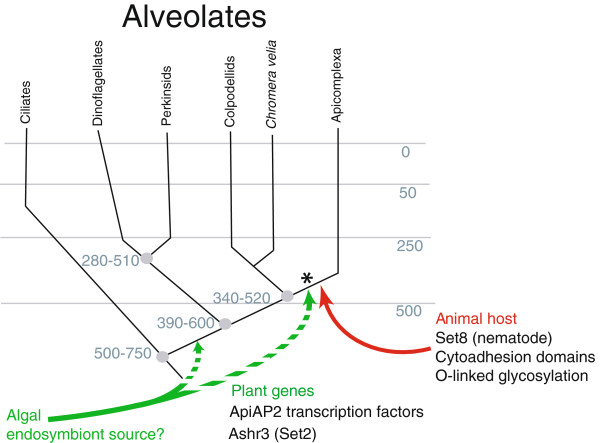
**The timing and source of horizontal transfer of genes that facilitate parasitism. **The evolution of alveolates is shown, with approximate times (million years ago) of major branching events denoted in gray type. Ancestral apicomplexa are thought to have appeared during the transition from the Neoproterozoic to the Paleozoic era, a time when nematodes and arthropods were extant, providing a possible source for transfer of Set8, cytoadhesion protein/invasion proteins and enzymes involved in O-linked glycosylation (red arrow). The proposed acquisition of the histone modifier Ashr3 and ApiAP2 transcription factors also appear to have occurred at or near this point in evolutionary history. The acquisition of an algal endosymbiont that is thought to be a source of ApiAP2 proteins and possibly Ashr3 is more controversial (dashed green arrows), but could also have coincided with the divergence of apicomplexan parasites. The apparent convergence of these distinct events within a narrow evolutionary window suggests that this represented a key moment in the development of apicomplexan parasitism (denoted by asterisk). Adapted from: Okamato, N. 2008. The mother of all parasites. Future Microbiology 3:391–395.

The observation that extant malaria parasites are capable of internalizing and incorporating exogenous host DNA spontaneously in animal cells is consistent with a potential mechanism of transfer [6,30]. Of special note is the observation that two other instances of horizontal gene transfer from animals are theorized to have occurred at a similar time in the evolution of these parasites (that is, into ancestors of modern apicomplexans). Domains involved in cytoadhesion and invasion, as well as genes required for O-linked glycosylation (GDP-fucose protein O-fucosyltransferase 2), are both proposed products of HGT dating from this time [21]. Notably, these are also missing from *P. marinus*, the nearest available relative of apicomplexans, consistent with a proposed HGT coincident to the Set8 transfer. The animal sources of the cytoadhesion domain and O-linked glycosylation HGTs, however, remain unidentified [7,31]. Although sampling of appropriate genomes remains relatively sparse, these results, taken together, suggest that during the transition to parasitism, in addition to acquiring the adhesion and glycosylation capabilities that underlie many of the intercellular interactions required for invasion and survival within their hosts, apicomplexan parasites also acquired additional transcriptional regulatory capabilities. These include both the ApiAP2 class of transcriptional regulators and the proteins needed for the epigenetic control utilized in cellular differentiation pathways and immune evasion. Moreover, these acquisitions appear to have occurred through HGT events at a similar time in evolutionary history, marking a key point in the transition of apicomplexans from a free-living to parasitic lifestyle (Figure 5).

Similarly, the ancestor of modern soil-based nematodes (e.g. *C. elegans*) could have transferred Set8 to the immediate ancestor of social amoebae and slime molds*.* Set8 orthologs are found in additional colonial slime molds (e.g. *Polysphondylium pallidum)* but are missing from other amoebae. Thus, the most parsimonious solution is an animal-derived HGT specific to slime molds. *Caenorhabditis elegans* is known to feed on *Dictyostelium,* ingesting and dispersing *Dictyostelium* spores [32]. This suggests that the incorporation of genes encoding nematode epigenetic machinery into the slime mold genome also is biologically feasible. Moreover, slime molds are known to adapt to predation pressure exerted by nematodes. This predation could provide a source of strong positive selection on enhanced control of gene expression in slime molds [32]. As evidence of the utility of DNA transfer, there are 16 known cases of horizontal gene transfer from bacterial sources into *Dictyostelium*[33,34]. Slime molds are well known to scavenge bacteria from the soil and recent reports indicate that bacteria are consumed by slime molds and incorporated into slime mold fruiting bodies and spores during asexual reproduction [35]. Thus it appears that over the course of evolution, slime molds have acquired DNA from both their predators and prey.

Notably, while the phylogenetic analyses described here suggest horizontal acquisition of both Set8 and a Set2 functional analog at or near the origin of the apicomplexan clade, there is significant flexibility in the requirements and functions of these proteins in various extant organisms. This mirrors and extends our earlier investigations on transcriptional machinery in *Plasmodium*. We recently identified an expanded motif within the tail of the RNA polymerase II CTD that is only otherwise found in animal (specifically, mammalian) host polymerases [2]. Intriguingly, this expansion is present only in primate malaria parasites and is absent in parasites of rodents and birds. In model eukaryotes, the CTD is connected to epigenetic memory through direct recruitment of SET-bearing methyltransferases that mark chromatin during active transcription [18]. Further demonstrating the flexibility in this aspect of transcriptional regulation, ‘Set2’ (Ashr3) is present in *Cryptosporidium,* whereas JmjC1 is absent, raising the intriguing possibility that they did not act together initially.

In *Plasmodium* specifically, the presence of additional RNA polymerase II CTD heptapeptides in primate parasites, a presumed requirement for recruitment of ‘Set2’, suggests that a cooperative interaction between ‘Set2’ and JmjC1 could have co-evolved with expansion of the CTD in these organisms. Consistent with this hypothesis, *Plasmodium* species that parasitize rodents do not have an expanded CTD and genes encoding Set2 and JmjC1 are missing from their genomes. Moreover, the deletion of both Set2 and JmjC1 in rodent plasmodia suggests tight and specific association between these chromatin modifiers in *Plasmodium;* this association is consistent with a recent integration into the genome, rather than with ancient, conserved functions that would have been harder to lose. The absence of epigenetic memory at rodent parasite surface-exposed variant antigens [36] (best studied in the *yir* family *in P. yoelii*) suggests a functional consequence of this deletion.

Our analysis also represents the first report of HGT of proteins involved in epigenetic control from animal provenance into slime molds. It is worth noting that among the amoebae, multicellular development and complex cellular communication and differentiation are found only in slime molds, the group that also acquired Set8 from animals [35,37]. We do not argue causality but simply raise the point that acquisitions of epigenetic machinery accompanied the evolution of higher order cellular processes in both apicomplexan parasites and social amoebozoans. Therefore, our work suggests that synergies between transcription machinery and epigenetic modifiers are at the nexus of the evolution of complex intracellular interactions, be they multicellular development or host/parasite interactions.

## Conclusions

We hope this work will shed light on the curious fate of lateral transfers from animal hosts into apicomplexans and other organisms, particularly SET-bearing chromatin modifiers and those molecules involved in epigenetic memory and immune evasion. Further investigations could reveal other novel, underappreciated acquisitions by protists at the dawn of apicomplexan parasitism that were functionally exploited as the immunological arms race between animal hosts and their parasites intensified. Targeting these factors that accompanied the transition to apicomplexan parasitism may prove crucial for advances in efforts to disable parasitism.

## Methods

### Identification, curation and phylogenetic analysis of Set8, Set2 and JmjC1 orthologs

The queries from canonical orthologs of Set8 from *Homo sapiens* were used in BlastP searches of the NCBI non-redundant protein databases for sequences from the Apicomplexa, dinoflagellates, ciliates, gregarines, diplomonads, rhodophytes, plants, Fungi, Microsporidia/Encephalitzoa, kinetoplastids, *Entamoeba,* heterokonts/stramenopiles, Amoebozoa (*Dictyostelium*), and various metazoan taxa including sponges, nematodes, trematodes, arthropods, trochozoans, echinoderms, birds, reptiles and primates. NCBI accession numbers for both query and retrieved sequences used for this and other phylogenetic analyses are included on the respective tree figures in this paper.

Comparative evolutionary analyses were carried out on all sequences using the following approach. Sequences were aligned using the program MUSCLE [38] and trimmed to include only the core SET domain that could be aligned clearly across all taxa included in the analyses. Phylogenetic relationships among sequences were resolved by both Bayesian inference (MrBayes) [39] and maximum-likelihood (PhyML) [40]. Parameters for phylogenetic analyses were determined using ‘Model Selection (ML) in MEGA 5.05 [41], and found to be the WAG substitution matrix and a gamma + I model for rate variation across sites (estimated from the data in both ML and Bayesian analyses) for all alignments used in this study. Both ML and Bayesian inference resulted in the same basic tree topologies in all analyses, with only minor rearrangements in sub-clades unrelated to the well-supported position of apicomplexans within the trees. The ML topology is shown in all tree figures. Relative support for tree nodes was inferred from Bayesian posterior probabilities estimated from all trees sampled once the average standard deviation of split frequencies had converged on a stable value, as well as through 100 nonparametric ML bootstrap replicates.

Comparable analyses were carried to test the phylogenetic affinity of *Plasmodium* Set2 and JmjC1 sequences. In these cases, we sampled NCBI protein databases with exemplars from both human and budding yeast, as well as sequences from *P. falciparum* and *T. gondii*. If different proteins were recovered using the alternative search strategies, both were included in initial respective phylogenetic analyses. For global phylogenetic analyses of SET domains, exemplars from model organisms (animals, yeasts and higher plants) were chosen to define established SET protein subfamilies. The ciliate and *Perkinsus* sequences included were the closest match to both Set2 exemplars from model eukaryotes and to putative ‘Set2’ homologs from the two apicomplexans. Because results from both search strategies were comparable, sequences were assembled into a single alignment for phylogenetic reconstruction. Since different closest matches tended to emerge in blastp searches using apicomplexan versus human and yeast JmjC1 queries, separate phylogenetic analyses were carried out on the two data sets. With human and yeast queries, apicomplexan JmjC1 tended to group with various green algal sequences rather than with *Perkinsus* or ciliates, their nearest alveolate relatives with complete and well-annotated genomes. In contrast, querying with apicomplexan sequences resulted in trees that grouped the apicomplexans and ciliates together. The latter were considered to be the most conservative estimates of JmjC1 sequence relationships and, therefore, were included in as our primary phylogenetic analyses (see results).

## Competing interests

The authors declare no competing financial, political, personal, religious, ideological, academic, intellectual, commercial or any other interests related to the work described in this publication.

## Authors’ contributions

SPK, JWS and KWD designed the study. SPK identified and assembled the datasets for Set2, Set8 and JmjC1 sequences. JWS conducted the maximum-likelihood and Bayesian inference analyses and generated the phylogenetic trees. SPK, JWS and KWD wrote the paper. All authors read and approved the final manuscript.

## Supplementary Material

Additional file 1: Figure S1 Expanded phylogenetic analyses of Set domain containing proteins showing that all putative Set2 (Ashr3) homologs from apicomplexans are recovered as a monophyletic group. Click here for file
