# Supplementary material for: Horizontal gene transfer of epigenetic machinery and evolution of parasitism in the malaria parasite Plasmodium falciparum and other apicomplexans
Source: BMC Evol Biol. 2013 Feb 11;13:37. doi: 10.1186/1471-2148-13-37 (PMC3598677; doi:10.1186/1471-2148-13-37)

Figure S1. Broader phylogenetic analyses of Set domain containing families, including putative 'Set2' homologs from all apicomplexans. Apicomplexan sequences are recovered as a strongly supported, monophyletic group. Moreover, they branch as sister to plant ASHR3 homologs, although with weaker support than in analyses with more balanced taxon-sampling (see Figure 3 in main article), rather than within a separate clade that contains canonical Set2 homologs from animals, plants and yeasts, as well as nearest homologs from ciliates. This topology is most consistent with horizontal transfer of an ASHR3 plant into the common ancestor of extant apicomplexans. Bayesian posterior probabilities and ML bootstrap support values are provided for relevant nodes of the tree.

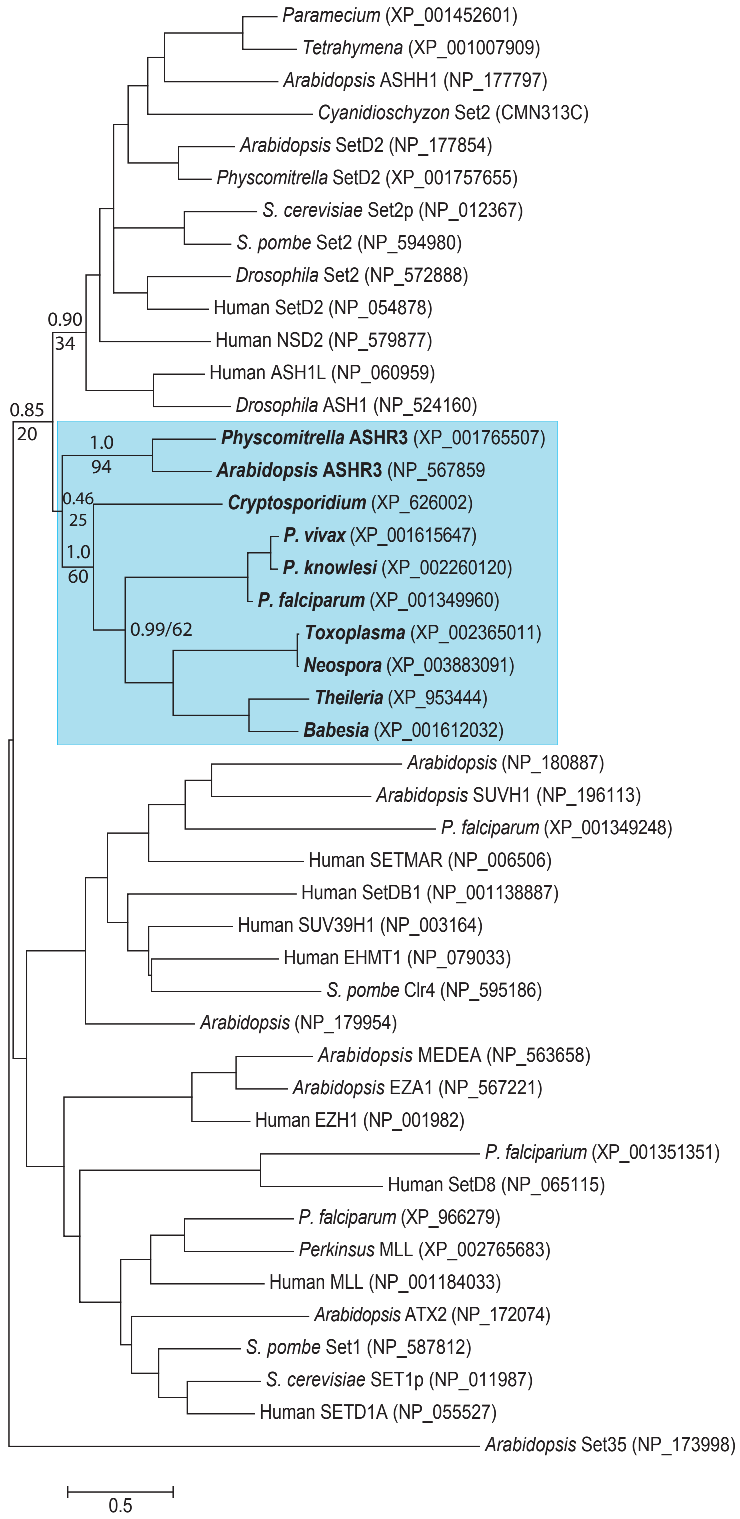

Supplement: Additional file 1: Figure S1 — Expanded phylogenetic analyses of Set domain containing proteins showing that all putative Set2 (Ashr3) homologs from apicomplexans are recovered as a monophyletic group. [file 1471-2148-13-37-S1.pdf]
